# Dr. Ferguson, on the Temperaments
*This was originally addressed to a Medical Gentleman of Aberdeen.


**Published:** 1809-03

**Authors:** 


### Dr. FERGUSON, on the TEMPERAMENTS.*

WE perceive in the human species great diversity of faces and character; and we find this difference not confined to their physical, but also applying to their moral Temperament. This difference was early known to the ancients, to the philosophers as well as physicians: but it more particularly came under the province of the latter, and they soon began to distinguish this difference by names. They considered certain circumstances being combined together in particular persons, and not in others, as forming different constitutions. This they supposed to originate from certain causes, therefore they named them temperaments. The appellations by which the temperaments are distinguished have been long in use, notwithstanding that their existence have been denied altogether, and the theories upon which they were founded long since exploded. It is not therefore surprising, that this branch of physic has long remained in an imperfect state, when we consider how many revolutions have happened in the theory and practice of physic since the days of Hippocrates. As we advance in our knowledge of the human system, of the principle of life, and of the morbid state, we will be better enabled to distinguish and judge of the temperaments, than we can do at present; and I am, Sir, of your opinion, that very little satisfactory and scientific knowledge is to be found on this intricate part of the animal economy.

To treat this subject properly would require great talents, and an intimate acquaintance with physiognomy as well as physiology. Since the humoral pathology has been exploded, and the system of life better understood; since our acquaintance with the circulation, the absorbent, secerning, lymphatic, and nervous systems, our views of medicine have been completely changed. The nervous is the grand system which moves the whole machine, and on the state of the fibrous motions of this depends our physical and moral temperament, health, predisposition, and disease.

It has been the general practice of physicians, to combine the theory of medicine with the temperaments; was this conducted upon a rational analogy, it is to be approved

 of;

* This was originally addressed to a Medical Gentleman of Aberdeen.

of; but when the choleric, phlegmatic, sanguine, and melancholic temperaments, are said to be occasioned by a humid and dry, hot and cold constitution, is it not sufficient to produce risibility in any reasonable philosopher?

As the action of heat and cold upon the human constitution is more rationally accounted for than formerly, and as we are better acquainted with the effect of every other agent which acts upon our system, our views therefore of many phenomena, which were considered as very mysterious, are now sufficiently elucidated. It will be obvious, that what occasions the difference of temperaments among mankind, is the same as produces difference of diseases. Where are we to look for the cause of this difference? Will it be found in the medullary and muscular fibre? Are we to look for it in that property or living principle, which, by the action of certain agents, produces all the phenomena of life? Yes. And from this state of its different faculties we are not only to look for a difference of temperament, but also disease.

This property has received different names by physiologists.—It is the excitability of Dr. Brown; the sensorial power of Dr. Darwin; the inherent power of Dr. Cullen; the vis insita, irritability, and vitality of others.

All the motions and sensations of our system depend upon it; and its excess or deficience is considered by Dr. Brown as the cause of disease. It is excited into action by different agents; these acting upon the organs of sense and muscular fibres, produce the excitement of the same author, or the sensorial powers of Dr. Darwin, which he terms irritation; or pleasure, or pain, when they excite into action the sensorial power termed sensation; and desire or aversion, when they excite into action the power of volition; or association, when any particular fibres are excited into action by preceding fibrous motions.

Dr. Darwin, therefore, has distinguished the temperaments into four kinds, agreeably to his theory of four classes of diseases, originating in the exuberance, deficiency, or retrograde action of the four faculties of the sensorium as the proximate cause, and in the disordered fibres of the body as the proximate effect of the exertions of those disordered faculties. His first temperament proceeds from a defect of sensorial power, and of course a torpid state of the system; or an accumulation of excitability, according to Brown, is the consequence: In the others there is an excess of sensorial power. They are

P 4 called, called, I. The temperament of decreased irritation. II. Of increased sensibility. III. Of increased voluntarity. IV. The temperament of increased association.

We meet with such diversity of temperaments among mankind, that it is difficult to arrange them under classes. This has been exemplified in the distinctions of the ancients; and, however simplified they may appear by the theory of Dr. Darwin, yet temperaments will be met with which it is not possible to rank under any of his classes. "There are not two individuals of the human species that have the same traits, who think precisely in the same manner, who see things in the same point of view, who have the same ideas; nor, in consequence, the same system of conduct. The visible organs of men, as well as their concealed organs, have indeed some analogy, some general points of resemblance and conformity, which make them appear in the gross, to be affected in the same manner by certain causes, but their differences are infinite in the detail."

It is in youth that our particular temperament is first formed, and we consider it as firmly established at the age of thirty. In childhood, neither the temperament nor the predisposition can be sufficiently distinguished; but, it is probable, that predisposition to certain diseases may exist at a very early period. In our northern climate we frequently find young children very vigorous, owing to their being reared in a hardy manner, and much exposed to a cool and pure air. And, what not a little contributes to their healthy and vigorous frame, is their being born of wholesome and pure blooded parents; for it may be said, with propriety, that in our mother's womb we draw together "the matter that shall, during our whole life, have an influence upon our intellectual faculties; upon our energies; upon our passions; upon our conduct." And Dr. Cullen is of the same opinion, when he observes, that the state of the simple solids, as well as the moving powers, depend much upon the original stamina.

The same author thinks the circumstances distinguishing the temperaments, may be referred to five general heads, according as they occur.—1st. In the state of the simple solids. 2d. In the state of the fluids. 3d. In the proportion of the solids and fluids. 4th. In the distribution of the fluids; and 5thly, In the state of the nervous power.

The temperaments, he observes, are not to be distinguished by attending to any one of these circumstances alone; alone; for the state of any of these is commonly combined with a particular state of all the others. A particular state of the simple solids is combined with a particular state of the fluids, with a particular state of the distribution and proportion of these, and all these with a particular state of the nervous system. Were these states steadily formed, and could they be perceived and distinctly marked in different persons, we might be enabled to form a doctrine of temperaments, free from embarrassment and complication. Are there not a great variety of different diseases, and yet we can distinguish them without the smallest difficulty? The reason is, their phenomena are distinctly marked; therefore, till the appearances of the different temperaments are more distinctly marked; the difficulty of distinguishing them will still continue.

Dr. Cullen considers the sanguine and melancholic temperaments, as the two that are most distinctly marked; and I find you are of the same opinion. His description of the sanguine, will apply better to the female character in general, or to youth, than to manhood; to weakness, rather than strength. As an opposite to this is the melancholic temperament, which he considers as occurring in the after parts of life, and consists of a greater density and less flexibility of the simple solid, with a proportional greater density and less mobility of the nervous power. These are the two temperaments which he says we can most clearly distinguish, and that different varieties are formed from these by the intermediate degrees of density in the simple solid and corresponding nervous power. In this way is the choleric and phlegmatic formed; in the former there is more strength than in the sanguine, and greater irritability than in the melancholic. In the latter, phlegmatic, less sensibility and irritabilty, but more strength and steadiness than in the sanguine, and greater laxity and more mutability than in the melancholic.

Thus far have I considered Cullen, and do confess, that the practical application of temperaments includes but few diseases. The sanguine are liable to hæmorrhagy, inflammation, and hysteria. The melancholic, to melancholia, hypochondriasis, melæna, and hæmorrhois.

Dr. Darwin's theory is rather upon a more extended scale in this respect, as it embraces his four classes of diseases.

You observe, "that in all those states of the system, where specific contagion or effluvia gives rise to small-pox-measles, angina maligna, chin-cough, syphilis, &c. we are soon bereft of any benefit to be drawn from this doctrine of of temperaments in the cure of these disorders; for instance, supposing two men, of different temperaments, to be attacked with the same complaint, perhaps typhus fever, or the yellow fever, each in the most violent and malignant degree: how soon would these two temperaments be confounded and lost amidst the virulence of the increasing symptoms of derangement in all the functions, so as to puzzle even Cullen himself, to draw any useful practical inference from them in cutting short or curing the disease? In those disorders too of the chronical type, such as ascites, diabetes, &c. how little light would this doctrine give us in the indications of cure."

Until we are better acquainted with the action of contagion (I speak of that kind which occasions malignant fevers) we cannot say with precision, what temperament either favours or tends to resist its influence. But there are some particular temperaments, that are more violently affected with contagion than others, and some particular constitutions, that have been known to resist even the attacks of the plague. Fat persons, in temperate climates, have escaped contagious fevers when they have been epidemic. And lean persons, in warm climates, have escaped, when those who were full and corpulent have quickly fallen a sacrifice to them.

Experience teaches us, that after we have been once affected with small-pox, measles, chin-cough, angina maligna, and some other species of fevers, the system resists their contagious influence in future. How are we to account for this? We cannot discern any change upon any of the functions, except a few remaining marks upon the skin; and yet we are fully satisfied a temperament is produced which secures us from their repetition. And since the discovery of the vacciniola of Jenner, a state of the system is induced, which prevents the infection of small pox. Do we not also find that the producing a mercurial action in the system prevents fever? But future experience must decide how far the knowledge of the temperaments are to teach us to regulate our indications of cure in the beginning, height, or decline of disease. When we are called to a patient, if we know his particular temperament, it will be of most service in the beginning of his disease; for when the disorder is actually formed, I agree with you, that the temperament will be then confounded and lost amidst the virulence of the increasing symptoms. Does not disease often change one temperament into another? And is not this often a cure of chronical disease?

The

The more I think on this subject, the more I am impressed with its importance, and I find that the most celebrated physicians have been of the same opinion. When the theory and practice of medicine are established upon a sure foundation, which can only render it eminently useful to the physician, the doctrine of the temperaments will be no longer imperfect, unintelligible, or embarrassed, the light thrown upon the one will be reflected by the other. I shall now offer a few observations for your consideration: Cullen, and all the modern writers upon this subject, consider the nervous power as modifying the temperaments of men, and upon its state depends the state of all the other parts. The brain is the organ which is the seat of nervous power and mental action.—It is acted upon by our sensual motions, and its own functions, which give sensation and motion to all parts. It appears, therefore, that all our motions are derived from the action of the brain, which is peculiarly organized for the purpose, and which communicates a contractile power to the muscular system. This contractile power is the principle of all animal motion. It is excited into action by desire or aversion, which is termed volition. If our pleasures and pains depend upon the imagination, certainly our volitions are much connected with it; and some are of opinion, that judgment, memory, and reason are only modifications of this. "The imagination perceives, reasons, judges, penetrates, and dives into things. The finest, the greatest, and the strongest imagination, is therefore the properest for the sciences as well as arts." What would the man of science, the orator or poet, be without it? A certain quantity and strength of it are necessary for the comparing the analogy or resemblance of ideas. The power of the imagination in marking the various relations of the qualifications useful to ourselves and others, give occasion to volitions; therefore, the operation of the imagination in marking the fitness or unfitness of things, and difference of this operation, with a quicker or slower perception of relations, are the distinguishing characters of the temperaments of men. It appears, therefore, that the temperaments depend upon the condition of the imagination, which condition is different upon certain occasions in the same person. The facility of renewing our ideas, and the degree of reflex sensation, or pleasure and pain attending them, are owing to the state of the imagination. Is it not by the imagination that we can combine our ideas; that we can acquire sensations of relation; that our our ideas are renewed by our memory; that we can compare our ideas; and that our sensations are modified?

"Imagination is the chief faculty employed in description, invention and persuasion, and in forming the various opinions by which mankind are governed. These opinions or imaginations, are confirmed by habit; and when erroneous, are not corrected even by experience. He that has most imagination, ought to be regarded as endowed with most wit and genius." There is certain conditions of it necessary for the formation of orators, musicians, painters and poets, and a different condition for the philosopher.

Although the imagination be weak in some, yet it is very impetuous and quick in others, especially in young persons, where it requires to be bounded by study and experience; it seems also to be dependent on the energy of the brain, organs of sense, and excitability. Is it not evident, that where the imagination is strong, there is vigorous excitability, vivacity, understanding and knowledge? Persons of this temperament have large brains and strong nerves; they are, however, very liable to be affected with particular pains and stimuli. The sanguine and choleric temperaments of the ancients, seem to be varieties of this. The opposite of the above temperament is attended with a dull imagination; the brain is small and the nerves slender. This agrees with the phlegmatic temperament of the ancients; the excitability is languid, ideas are defective, and the judgment weak. Persons of this temperament are more able to bear fatigue and hardship of every kind, than those of the quick temperament. Of these two we may consider all the temperaments to be formed, those of debility, as well as those of strength, the sanguiue, the melancholy, the phlegmatic, and choleric.

We may consider the strength of the system to be regulated by the functions of the brain; for where there is a quickness of imagination, there is a facility of performing voluntary motion, a stronger energy of the brain, and a more vigorous excitability, or that facility of muscular contraction, termed irritability.

I perceive Mr. Humbold thinks it "highly probable, that the property of matter to be affected by stimulus, depends upon its composition, and that every thing altering this, modifies its excitability; and he therefore concludes, that nothing is of itself, either stimulating or sedative, and that the action entirely depends on the state of the organs with which it enters into combination; and that the great process process of life consists in a perpetual alteration of decomposition and union, and that substances arbitrarily added to, or abstracted from living matter, sometimes diminish, and sometimes increase the activity of the organs." It is, therefore, very probable that the different proportion and combinations of elasticity and density, may modify the excitability. We must however remain in a state of uncertainty, with respect to that peculiar state of the organs of sense and motion, which modifies the vitality and temperament, until, as you observe, we are better acquainted with the chemical changes, which happen during the functions of life. But let us proceed with our observations on the particular temperaments.

I should suppose in the temperament of vigorous imanation, where the sensibility is in its utmost perfection, that the functions of the brain were performed with great regularity, that the secretion of the nervous fluid was in due proportion and of proper density, that the vis nervea was of sufficient force, and obeyed the volitions of the imagination, and that the excitability was vigorous: in consequence of this, I should suppose the judgment to be strong, attended with greatness of mind, and those qualities agreeable to ourselves to be predominant, as knowledge, cheerfulness, courage, tranquillity and benevolence. "Nor should I suppose this character destitute of those qualities considered agreeable to others, as justice, good manners, wit, and ingenuity. Does he not esteem justice, fidelity, honour, veracity, allegiance, and chastity, on account of their tendency to promote the good of society? And is not that tendency inseparable from humanity, benevolence, lenity, generosity, gratitude, moderation, tenderness, friendship, and all the other social virtues? Nor can it be doubted that industry, discretion, frugality, secrecy, order, perseverance, fore-thought, judgment, and the whole class of virtues and accomplishments, of which many pages would not contain the catalogue: can it be doubted, I say, that the tendency of these qualities to promote the interest and happiness of their possessor, is the foundation of their merit?

Who can dispute that a mind, which supports a perpetual serenity and cheerfulness, a noble dignity and undaunted spirit, a tender affection and good will to all around, as it has more enjoyment within itself, is also a more animating and rejoicing spectacle, than if dejected with melancholy, tormented with anxiety, irritated with rage, or sunk into the most abject baseness and degeneracy?

And

And as to qualities immediately agreeable to others, they speak sufficiently for themselves; and he must be unhappy indeed, either in his own temper, or in his situation and company, who has never perceived the charms of a facetious wit or flowing affability, of a delicate modesty or decent gentleness of address and manner." This temperament we have supposed to be of the most finished cast, possessing the most enjoyment and the least suffering, with the most perfect mental organization, free from imbecility; with a spacious forehead, well defined features, and a large brain. Of a healthy and fresh colour, with union and harmony in the form. The expression of the countenance, indicative of the state of the mind, having a beautiful serenity, or a mixture of modesty, sensibility, and sweetness. Nor should we suppose the following signs of force and vigour absent, viz. "Broad shoulders, a lank belly, firm joints, and taper legs." This is the temperament most capable of moderation, and of doing most good, and will serve to answer the four following queries. "Which is the temperament most capable of friendship? Which is happiest united in marriage? Under what temperament do men live longest?" and, Which is most free from disease? Some will observe, that there are very few who enjoy such a happy temperament as I have described; I rather suppose, that there are a great many of this temperament, and that there would be more, if it were not owing to ambition, avarice, and intemperance. The times in which we live, require the greatest diligence and œconomy, to procure a respectful appearance, and supply our wants, which, from our increased desires, are become many. Professional men are eager in the pursuit either of fame or riches. Commerce gives such a bent to the mind, that is often productive of the worst consequences.

There are so many different scenes to be met with in fashionable life, which affect the mind in such a manner, that it is no wonder when they excite in us the most destructive passions, and enervate our mental functions; they are too frequently productive of luxury and dissipation, which lead to follies, absurdities, and crimes.

We are not therefore to expect that we shall often meet with this temperament among professional men, nor the commercial world in general. We ought to meet with it where the wants are least, especially among those who enjoy a happy retirement from the busy scenes of life; who neither indulge in luxury nor dissipation, but enjoy a calm calm domestic agricultural life. May we not expect to meet with it in clergymen?

The opposite temperament to this I should suppose to consist of a small brain with slender nerves; the imagination less vigorous; the excitability either more languid or exhausted; less sensibility; ideas more defective; judgment weaker; memory untenacious; and greater irregularity and weakness of the mental functions; we are not therefore to expect those virtues and qualities agreeable to ourselves and others, to be so eminently displayed as in the former temperament; but on the contrary, there must be less enjoyment and more suffering, because, instead of those duties referable to probity and justice, there is nothing but malice, folly, fear, sensuality, and dissipation.

The effects of a dull imagination will be evident in the countenance, which is the index of the mind; the condition of our mental faculties are represented there, and every passion and mode of thinking has its peculiar expression. We can be at no difficulty in distinguishing when a man looks upon us with pleasure or pain; or where there is a deficiency of ideas, by the vacant and unmeaning aspect.

Nor is it to be expected that in this temperament there should be such a beauty or uniformity in the appearance. "The body and mind have such mutual influence, that whatever contributes to change the human constitution in its form or aspect, has an equal influence on its powers of reason and genius. And these again have a reciprocal effect in forming the countenance."

Thus have I considered the two classes of temperaments, depending upon the state of the imagination and mental faculties, which diversify to infinity, the differences that are to be found among men. It is to this that some are called good, and others dull, rustic, choleric, melancholic, phlegmatic, cruel, malicious, and dissipated; wicked, virtuous, and vicious; learned and unlearned; reasonable and unreasonable. There is a certain balance of the mental functions which is necessary to and constitutes the first class of temperaments; this, when properly supported, gives energy to the nervous system; but if by any means this balance is broken, either by intemperance or excess of the passions, predisposition and disease are the consequence. The same balance gives to the mind its share of good and evil, and is also necessary to the excitability of the system and action of the brain. The imagination in excess may, therefore, be the means of destroying this balance; balance; and a permanent predisposition be formed to mania, fever, and inflammation. This is similar to the temperament known by the name of choleric. There is often a degree of energy and ferocity in the countenance; small eyes and contracted eye-brows; the actions of the muscles quick, especially in walking and speaking; much inclined to dreaming; fond of fermented liquors and animal food; eager to undertake hazardous enterprises, and very desirous of commanding.

When the imagination is weak, with deficiency of excitability and sensation, this is always accompanied with moral weakness, and corresponds with the melancholic temperament; there is a languor of the countenance, the muscles of the eye-lids and brows hang down, and give a dejected appearance to the face; the hair black; they bear pain better than labour; are much affected by the changes of the atmosphere, and are disposed to have cold feet; their, volitions are slow; although they are very attentive, yet they are slow to learn; they submit to labour with patience, and are very frequently passionate and revengeful. Those of this temperament are subject to hysteric affections, spasms, hæmorrhagies, nervous fevers, and the other diseases of debility.

The hypochondriac is a variety of this temperament; they have been supposed to be very subject to diseases of the liver; at any rate they are very whimsical, discontented, and envious. God forbid that any magistrate should be of this temperament. From the different mixtures of the quick and dull temperaments, all the others may be supposed to be formed; the mild; the rustic; the female, &c. The female temperament may be supposed to differ in several respects from the male. They are formed for giving pleasure, and are therefore tender, delicate, and affectionate, but weaker than men. There is also a difference occasioned by the uterine and mammillary systems, which have a very great effect upon their excitability. Although they have not such a depth of thought as the male, their sensibility is greater, and they have a very quick conception of things. Their passions are for the most part strong, and they have not the same power over them as the male; they are very credulous and enthusiastic, and yet their affection and love are more durable than the male; they are also very patient, benevolent, and modest, but are very easily sunk into melancholy, or raised to rapture. I observe that novel writers make their heroines very liable to syncope upon very trifling occasions. Is this a characteristic of the female temperament? I believe they are more permanently predisposed to the diseases of debility than the male.

*Aberdeen-Green, October* 18, 1808.

